# Astroglial Modulation of Hydromineral Balance and Cerebral Edema

**DOI:** 10.3389/fnmol.2018.00204

**Published:** 2018-06-12

**Authors:** Yu-Feng Wang, Vladimir Parpura

**Affiliations:** ^1^Department of Physiology, School of Basic Medical Sciences, Harbin Medical University, Harbin, China; ^2^Department of Neurobiology, The University of Alabama at Birmingham, Birmingham, AL, United States

**Keywords:** astrocytes, cerebral edema, osmosensation, osmotransduction, vasopressin

## Abstract

Maintenance of hydromineral balance (HB) is an essential condition for life activity at cellular, tissue, organ and system levels. This activity has been considered as a function of the osmotic regulatory system that focuses on hypothalamic vasopressin (VP) neurons, which can reflexively release VP into the brain and blood to meet the demand of HB. Recently, astrocytes have emerged as an essential component of the osmotic regulatory system in addition to functioning as a regulator of the HB at cellular and tissue levels. Astrocytes express all the components of osmoreceptors, including aquaporins, molecules of the extracellular matrix, integrins and transient receptor potential channels, with an operational dynamic range allowing them to detect and respond to osmotic changes, perhaps more efficiently than neurons. The resultant responses, i.e., astroglial morphological and functional plasticity in the supraoptic and paraventricular nuclei, can be conveyed, physically and chemically, to adjacent VP neurons, thereby influencing HB at the system level. In addition, astrocytes, particularly those in the circumventricular organs, are involved not only in VP-mediated osmotic regulation, but also in regulation of other osmolality-modulating hormones, including natriuretic peptides and angiotensin. Thus, astrocytes play a role in local/brain and systemic HB. The adaptive astrocytic reactions to osmotic challenges are associated with signaling events related to the expression of glial fibrillary acidic protein and aquaporin 4 to promote cell survival and repair. However, prolonged osmotic stress can initiate inflammatory and apoptotic signaling processes, leading to glial dysfunction and a variety of brain diseases. Among many diseases of brain injury and hydromineral disorders, cytotoxic and osmotic cerebral edemas are the most common pathological manifestation. Hyponatremia is the most common cause of osmotic cerebral edema. Overly fast correction of hyponatremia could lead to central pontine myelinolysis. Ischemic stroke exemplifies cytotoxic cerebral edema. In this review, we summarize and analyze the osmosensory functions of astrocytes and their implications in cerebral edema.

## Introduction

Homeostasis of the internal environment is the prerequisite for normal activity of an organism and heavily depends on the hydromineral balance (HB) of the extracellular fluid. This balance is based on equivalent amounts of water drinking and salt intake vs. their excretion, and is commonly measured by the volume and osmolality of the extracellular fluid (Muhsin and Mount, 2016). Many factors have been implicated in regulation of HB, such as thirst, along with water- and salt-regulating hormones. Among hormones, vasopressin (VP, also called as antidiuretic hormone, ADH) released by hypothalamic neuroendocrine cells has been considered the most sensitive and powerful factor regulating HB (Kinsman et al., 2017). In response to increased osmotic pressure or reduced blood volume, VP release into the brain and blood increases significantly to surge reabsorption of water, thereby maintaining relative fidelity of the osmolality and volume of the brain and blood (Brown, 2016). However, maladapted response of this VP secretion can cause cerebral edema and threaten the life of patients, e.g., due to high intracranial pressure and the resultant brain herniation in hyponatremia (Wang et al., 2011) or ischemic stroke (Jia et al., 2016). Thus, understanding the mechanisms underlying neurohumoral regulation of VP secretion is critically important.

In neurohumoral regulation of VP neuronal activity, one of the most sensitive and important factors is astrocytic plasticity in the osmotic regulatory system (Wang and Zhu, 2014; Jiao et al., 2017). Astrocytes adjacent to neurons in the osmosensory system are sensitive to VP and can also influence VP secretion through their morphological and functional plasticity and produce other hormones that also regulate osmolality, while directly regulating local HB; of note, a variety of astrocytes in other brain areas are sensitive to VP (Simard and Nedergaard, 2004), a subject that is beyond the scope of the present review, however. Nonetheless, under pathological conditions, malfunctions of osmosensory mechanisms in astrocytes worsen hydromineral disorders by affecting the activity of VP neurons and other osmotic regulatory factors; these events lead to brain edema, neural degeneration and irreversible brain damages. In this review, we sum up our current understanding of astrocyte-associated osmosensation, osmotransduction and the resultant changes in the activity of the osmotic regulatory system, along with their implication in diseases involving disorders of HB.

## Osmotic Balance and its Neurohumoral Regulation

Homeostasis of the internal environment is under constant challenge of the external environment. Factors that can markedly change extracellular and intracellular fluid volume include ingestion and elimination of water and salts, hydromineral distribution across capillaries (e.g., within the blood vs. brain parenchyma/other organs) and, at the cellular level, across the plasma membrane. In response to these challenges, the endocrine system and the autonomic nervous system respond promptly to adaptively modulate the HB by regulating water and salts intake and elimination, as well as their distribution among different compartments of the body.

### General Regulation of HB

Hydromineral regulation requires activation of the osmosensory system in conjunction with volemic regulation and is largely determined by a variety of neurohumoral factors. An increase in plasma osmolality draws water from cells and interstitium into the blood, causing dehydration, which activates specific brain osmoreceptors to stimulate drinking and release of VP from VP neurons. VP reduces water loss via increasing water reabsorption in the kidneys. In contrast, hypoosmotic challenge causes opposite reaction by VP neurons and the hydromineral regulation. Hypovolemia, a state of decreased blood volume, stimulates vascular volume sensors that signal brain centers to initiate drinking and VP release; it also stimulates baro/volume receptors in the kidneys to activate the renin-angiotensin (Ang)-aldosterone system (RAAS), which initiates drinking and VP release while increasing Na^+^ reabsorption. By contrast, blood volume expansion or hypervolemia can lead to significant increase in plasma concentrations of atrial natriuretic peptide (ANP), oxytocin (OT), prolactin and corticosterone, to eliminate Na^+^ and its bound water while suppressing hyperosmotic stimulation of VP secretion. In addition, osmotic challenge can lead to the production of gaseous neurotransmitters, which can affect neuronal secretion; e.g., nitric oxide (NO) inhibits (Stern and Ludwig, 2001), while carbon monoxide stimulates (Reis et al., 2012) VP secretion. The aforementioned factors and events, and likely many more, all work in concert so that the HB is maintained.

To achieve electroneutrality and volume stability of a cell, the sum of osmotically active particles, i.e., osmolytes, in the intracellular space must be equal to that in the extracellular space. As the major extracellular osmolyte, Na^+^ in optimal extracellular concentration becomes the reference point for osmoreceptors to control thirst and VP secretion, RAAS activity, levels of natriuretic factors as well as the cell volume. The control over the cell volume is achieved by a chloride shift and by modulating the activity of Na^+^/K^+^-ATPase (Kurbel, 2008). Movement of water across the cell membranes occurs when an osmotic pressure gradient forms between the intracellular compartment and interstitial fluid. Large amounts of Na^+^, Cl^−^ and HCO_3_^−^ ions are present in the extracellular space whereas K^+^, Mg^2+^ and PO_4_^3−^ ions are the major ions in the intracellular compartment. In most cells at rest, the plasma membrane exhibits relatively high permeability for K^+^ and water, but not for Na^+^ and Cl^−^. Consequently, the distribution of fluid across the plasma membrane is determined mainly by the osmotic effect of Na^+^ and Cl^−^. Whenever osmotic gradients form, following disturbance of the electrochemical balance across the membranes, water will diffuse to the side of higher solute concentration, thereby maintaining intracellular fluid isotonic. The transport of ions and water across cell membranes has been reviewed recently elsewhere (Jia et al., 2016) and, hence, is not further discussed here.

**Figure 1:**
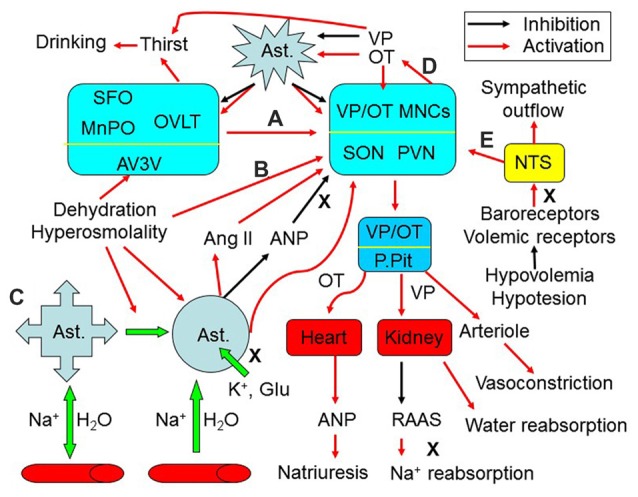
Extrinsic modulation of hydromineral balance (HB). The sketch shows that hyperosmotic stress modulates HB by the following approaches. **(A)** Activating the subfornical organ (SFO), organum vasculosum of the lamina terminalis (OVLT) and the medial preoptic nucleus (MnPO) in the anteroventral-third ventricle (AV3V) area, which in turn activates magnocellular cells. i.e., vasopressin (VP) and oxytocin (OT) neurons in the supraoptic (SON) and paraventricular (PVN) nuclei. **(B)** Changing activity of VP neurons after the osmolality is increased in brain parenchyma. **(C)** Causing retraction of astrocyte (Ast.) processes from the areas surrounding the osmosensory neurons, which increases the excitability of VP neurons by removing the physical barriers between adjacent neurons and by increasing extracellular levels of K^+^, glutamate (Glu) and angiotensin II (Ang II), and by reducing extracellular levels of atrial natriuretic peptide (ANP) or brain natriuretic peptide. **(D)** Resultant increases in intrahypothalamic release of OT can activate VP neurons through eliciting the retraction of astrocytic processes, while VP inhibits this retraction by increasing aquaporin expression. **(E)** In addition, hypovolemia or hypotension can increase VP release from the posterior pituitary (P.Pit) by reducing the baroreflex through the nucleus of the tractus solitarii (NTS) and by decreasing activity of the renin–angiotensin–aldosterone system (RAAS), while increasing the sympathetic output. As a result, serum osmotic pressure gets reduced and blood volume/pressure rises through increasing water reabsorption and natriuresis. See text for details.

Along with the effect on water balance, VP is also a critical modulator of the arterial diameter, which along with water reabsorption/distribution, determines the effective arterial volume. For instance, in the rat hypothalamic supraoptic nucleus (SON), hyperosmotic stimulation mainly led to vasodilatation followed by vasoconstriction (Du et al., 2015). The initial vasodilatation could be the result of both glial retraction and NO action at the blood vessels, while the later vasoconstriction phase results from VP-mediated activation of V1a VP receptor. By changing the arterial contractility and the blood pressure, VP can maintain irrigation pressure to provide sufficient nutrients to the brain and other important organs (Figure 1).

### VP Neuronal Activity and its Neural Regulation

VP is an essential regulator of the HB. In the hypothalamus, there are several neuroendocrine nuclei that contain VP neurons. These cells are primarily located in the SON and the paraventricular nucleus (PVN); they are mainly magnocellular neuroendocrine cells (MNCs) releasing VP into the blood and brain. In addition, there are also parvocellular VP neurons in the parvocellular section of the PVN that release VP to regulate the activity of the autonomic nervous system and a variety of brain activities (Hou et al., 2016). In patients with central diabetes insipidus, the lack of VP production causes thirst (polydipsia) and excessive excretion of urine (polyuria) because of a dysfunction in the thirst mechanism, development of hyponatremia and the ensuing complications (Sailer et al., 2017).

The effects of osmotic challenges on VP neuronal activity and VP secretion depend on both external factors and the intrinsic features of these neurons (Figure 1). Classically, neural regulation is considered the major factor modulating VP neuronal activity during osmotic challenges (Figure 1A). Brain regions around the circumventricular organs (CVOs) and the nucleus of the tractus solitarii (NTS) are the major source of neural inputs and regulation of VP neuronal activity (Wang et al., 2011; McKinley et al., 2015). The CVOs are highly vascularized brain areas lacking a normal/tight blood-brain barrier (BBB). These organs/structures include (but are not limited to) the subfornical organ (SFO), the organum vasculosum of the lamina terminalis (OVLT) and the area postrema. They are the sites allowing communication between the blood plasma and the interstitium of the brain parenchyma, as their fenestrated capillaries are freely permeable to ions and water. Neural inputs from the NTS can relay hypovolemic/hypotensive information to the CVOs while directly modulating VP neuronal activity to correct fluid deficits (Miyata, 2015). However, CVOs and the NTS do not regulate the HB directly; rather, they convey osmotic and volemic messages to VP neurons. Among the CVOs, the SFO can sense and integrate hydromineral signals in responses to osmotic, volumetric and cardiovascular challenges, and integrate information from circulating signals of the metabolic status (Hindmarch and Ferguson, 2016). Moreover, VP neurons can also be regulated by other brain regions involved in the integration of circulatory and fluid information (McKinley and McAllen, 2007). Activation of neurons in these brain areas, which include the lateral parabrachial nucleus, the midbrain raphe nuclei, the medial preoptic nucleus and the septum, increases VP neuronal activity in the SON and PVN, and VP secretion that modulates kidney functions and arteriole contractility, along with activation of the anterior cingulate cortex and insula to cause thirst and resultant water drinking (Macchione et al., 2015). The efficiency of the above neuronal inputs on VP neuronal activity depends on astrocytic plasticity and the neurochemical environment surrounding VP neurons (Wang and Zhu, 2014).

### Astrocytic Regulation of HB

In the extrinsic modulation of VP neuronal activity, astrocytic plasticity plays an important role in local and systemic HB (Wang et al., 2011). Astrocytes are the sensors of neuronal activity while detecting humoral information from the blood at the CVOs (Farmer et al., 2016). The responsive changes of astrocytes, i.e., their plasticity, in turn modulate VP neuronal activity, VP secretion and thirst.

Similar to the modulatory role of astrocytes in neuronal activity (Simard and Nedergaard, 2004), astrocytic morphological plasticity is also essential for the integrity of the neurovascular unit. Of note, the neurovascular unit is a functional unit comprised of capillary endothelial cells, along with surrounding neurons, and non-neuronal cells such as pericytes and astrocytes. Malfunction of astrocytes can change the structural integrity of this unit and subsequently disturb the HB across the BBB and the transport of metabolites, ions, varieties of organic molecules and water between the brain and the blood (Wang and Parpura, 2016).

#### Astrocytic Morphological Plasticity and VP Neuronal Activity

In osmosensation, astrocytes exhibit remarkable morphological plasticity (Figure 1C). It was observed that after exposing cell culture to a hypoosmotic solution, astrocytes swell within 30 s and then undergo a regulatory volume decrease (RVD; Eriksson et al., 1992) following the opening of anion channels and release of organic osmolytes. Conversely, when exposed to a hyperosmotic solution, astrocytes shrink first and then exhibit a regulatory volume increase (RVI; Eriksson et al., 1992). This dual morphological plasticity, occurring during acute hypo- or hyper-osmolality, provides astrocytes with unique capacity to keep the extracellular volume stable while differentially regulating extracellular osmolality. Regulatory volume changes could occur in a microdomain specific manner or as a result of intracellular volume redistribution; that is, an expansion of astrocytic processes with a reduced size of astrocytic somata and vice versa (Choe et al., 2016).

Different from the RVI and RVD mentioned above, when exposed to a gradual osmolality decrease, some cells use the mechanism of isovolumetric regulation to adapt to the hypoosmotic challenge without dramatically changing their volume. The apparent isovolumetry is due to the coordinated extrusion of osmolytes, whereby following an early efflux of taurine (2-aminoethanesulfonate^−^) and Cl^−^, a delayed K^+^ efflux occurs, as shown in cerebellar astrocytes (Ordaz et al., 2004). These diverse volemic regulatory machineries endow astrocytes with the ability to directly sense and regulate HB in local brain parenchyma.

An example of astrocytic involvement in osmotic regulation is the osmotic response of the SON during osmotic challenges. Plastic change in the morphology of the SON and PVN is a responsive feature to hydromineral disturbance, which is particularly dramatic in the SON during dehydration. As previously reviewed (Hatton, 2002), during dehydration or salt (hypertonic) water drinking, astrocytes show retraction of their processes along with hypertrophic changes in VP neurons. This retraction of astrocytic processes from the surroundings of VP neurons increases the interaction between neurons through interneuronal/dendritic gap junctions and preponderance of synaptic connections, thereby increasing the excitability of VP neurons and promoting synchronous release of VP. To the contrary, hypoosmotic challenges can inhibit VP neuronal activity by evoking the expansion of astrocytic processes, wedging them in between neuronal dendrites and lessening dendritic gap junctional connectivity. These changes reduce direct interactions between adjacent VP neurons, while increasing astrocytic uptake of extracellular K^+^ and glutamate; opposite effects are seen during hyperosmotic stress. Correspondingly, VP neurons become more excitable during hyperosmotic stress and inhibited during acute hypoosmotic challenges.

It is worth to notice that during chronic hyperosmotic stimulation, VP release remains at high levels. This is likely due to increased VP synthesis as well as increased release probability of VP packaged in secretory vesicles, along with increased OT-mediated excitation (Figure 1D). It has been reported that during chronic hypernatremia, VP and OT mRNA levels increase twofold along with hypertrophy of VP neurons and increased release of VP (Glasgow et al., 2000). Moreover, this condition also causes an activity-dependent depolarizing drift in the chloride reversal potential and abolition of inhibitory pathways, which is prevented by blocking central OT receptors (Kim et al., 2011), suggesting that hyperosmotic stress increased OT receptor signaling that is responsible for the reversal of GABAergic inhibition. Consistently, OT can excite VP neurons (Wang and Hatton, 2006) while exerting autoregulatory effect on OT neuronal activity (Wang et al., 2006; Wang and Hatton, 2007a,b). Thus, the maintenance of high level of VP release during chronic hyperosmotic stimulation is at least partially supported by an increase of OT release. Noteworthy is that the excitatory effect of OT on VP neurons takes minutes to occur, and thus is highly unlikely a direct effect on OT receptors on VP neurons, but most likely an indirect effect due to a retraction of astrocyte processes in the SON caused by OT (Wang and Hatton, 2009; Wang et al., 2017).

Astrocytes of the SON have the ability to change the osmosensory threshold within the local neural circuitry during chronic hypoosmotic challenges, as previously reviewed elsewhere (Wang et al., 2011). That is, the sensitivity of osmosensors to hypoosmotic challenge gradually wears off during continuous stimulation; correspondingly, the initial inhibitory reaction vanishes during prolonged presence of hypoosmotic environment. This notion is partly supported by the recovery of firing rate of VP neurons after initial hypoosmotic inhibition, which is based on dual astrocytic morphological plasticity, i.e., extension and subsequent retraction of astroglial processes during the initial decrease in firing rate of VP neurons followed by its recovery, respectively (Wang et al., 2013a,b).

Taken together, astrocytic morphological plasticity can modulate the activity of osmosensory neurons (OS neurons), which in turn changes astrocytic plasticity, thereby forming a functional heterocellular network in central osmosensation (Figure 1).

#### Astrocytic Functional Plasticity and VP Neuronal Activity

Accumulating evidence reveals that astrocytes are not just a passive buffer of extracellular environment, but also an active modulator of neuronal activity (Figure 1C). Astrocytic modulation of VP neuronal activity is closely related to its ability to take-up neurotransmitters from the extracellular space (Wang et al., 2013a,b) as well as to produce, release and take-up gliotransmitters to modulate interneuronal communication (Parpura et al., 2004; Montana et al., 2006; Ni et al., 2007). As the source of glutamate in the SON (Ponzio et al., 2006), astrocytes can excite OS neurons, including VP neurons during hyperosmotic stimulation. Moreover, the presumed reduction in uptake of β-alanine by astrocytes in the SON can inhibit GABA transporters and subsequently increase extracellular GABA levels (Park et al., 2006; Wang et al., 2013a). Therefore, astrocytes can actively alter the neurochemical environment and modulate the activity of OS neurons. Of note, cultured astrocytes from different brain regions can release various other gliotransmitters using multiple underlying mechanisms and in response not only to hypoosmotic challenge but also to an array of neurotransmitters and modulators (Malarkey and Parpura, 2009). Whether a palette of gliotransmitters, released by astrocytes elsewhere, could be released by astrocytes in the SON in times of osmotic challenge and whether that would lead to modulation of synaptic transmission in the SON remain to be determined.

## Astrocytic Interaction With Hormones That Regulate Osmolality

While astrocytes can directly regulate thirst and maintain the integrity of neurovascular units, they also directly interact with hormones that regulate osmolality, including VP, Ang II, aldosterone, natriuretic peptide, OT and hormones in the hypothalamic-pituitary-adrenal axis. Thus, astrocytes can modulate the effect of hormones on osmotic regulation of HB (Figure 1C).

### Astrocytes and VP

It has been reported that hyperosmotic stimulation increases VP release in the brain (Ludwig et al., 2005). VP in the brain can evoke thirst and drinking (Abrão Saad et al., 2004), while increased levels of VP in the blood increase water reabsorption in the kidney by changing water channel activity (Tamma et al., 2017). In addition, VP can antagonize diuresis and natriuretic role of ANP (Lipari et al., 2007), thereby limiting ANP’s natriuretic effect. As a result, VP is both an antagonizing agent of hypovolemia or hyperosmolality and a facilitator of hyponatremia; excess release of VP may result in water retention and pathophysiological hyponatremia.

VP is a major facilitator of cell swelling in the brain, which involves several local pathways. VP can increase intracellular osmolality by uptake of Na^+^, K^+^, 2 Cl^−^ or by lack of extrusion of Na^+^ via activating Na^+^, K^+^, 2 Cl^−^ and water cotransporter 1 (NKCC1) or via inhibiting the activity of Na^+^/K^+^-ATPase, respectively (Hertz et al., 2017). In addition, VP is also a major activator of astrocytic water channel protein, aquaporin 4 (AQP4) by activating V1a type of VP receptor (Niermann et al., 2001). Inhibition of VP1a receptor leads to decrease in AQP-4 expression and prevents brain edema after port-traumatic injury (Marmarou et al., 2014). Thereby, VP could facilitate cell swelling when intracellular osmotic pressure is higher than that of the extracellular fluid.

### Astrocytes and RAAS

Along with water reabsorption/excretion mediated by VP secretion, the sodium-retaining function of the RAAS makes a critical contribution to extracellular Na^+^ levels and osmolality. In the central nervous system, the RAAS acts mainly through the sensory CVOs, in particular the area postrema, to activate brain neural pathways that elevate blood pressure, release VP and aldosterone, increase renal sympathetic nerve activity, and increase the ingestion of water and Na^+^ to restore Na^+^ loss to the environment (Geerling and Loewy, 2008). In these processes, Ang II binds to the brain Ang type 1 receptor to stimulate thirst, Na^+^ appetite and secretion of VP and OT (Felgendreger et al., 2013; Almeida-Pereira et al., 2016). The prolonged Ang type 1 receptor blockade caused rebound increase in levels of Ang II and VP secretion to compensate for hypovolemia in association with reduced ANP plasma concentrations (Araujo et al., 2013). These findings are in agreement with the classical function of the RAAS in Na^+^ balance.

Astrocytes are a major source of the Ang II precursor protein angiotensinogen and Ang II in the brain (Hermann et al., 1988); expression of receptors for Ang was also identified in the rat and monkey astrocytes (Garrido-Gil et al., 2017). Thus, astrocytes could exert self- modulation during osmotic challenges. This notion is supported by the observation that following 7 and 14 days of 2% NaCl (N.B., isotonic solution contains 0.9% NaCl) in drinking water, a significant increase in Ang II precursor, preproangiotensinogen mRNA was detected in astrocytes in regions of the anterior hypothalamus, including the PVN, the medial preoptic area and medial preoptic nucleus, while a decrease was observed in astrocytes in the SON (Ryan and Gundlach, 1997). These results, consistent with a recent report (Dominguez-Meijide et al., 2017), indicate that astrocytes could increase levels of extracellular Ang II to regulate thirst, while Ang II facilitation of VP release is reduced during prolonged hyperosmotic stimulation.

### Astrocytes and Natriuretic System

In contrast with the sodium-retaining functions of the RAAS, natriuretic peptides, such as ANP, brain natriuretic peptide, and OT, belong to the “natriuretic system.” OT neurons in the hypothalamus can be activated simultaneously with VP neurons during dehydration or hypertonic stimulation. However, OT exerts a natriuretic function, which plays a synergistic role in maintaining HB with VP-increased water reabsorption following hyperosmotic stimulation. Similar to the modulation of VP neurons in hydromineral disturbance, astrocytic plasticity also strongly influences OT secretion from the SON and PVN (Yuan et al., 2010). In addition, during blood volume expansion, OT is secreted from the posterior pituitary into circulation to activate atrial OT receptors in the heart and promote ANP release. ANP diminishes VP-induced water reabsorption while exerting natriuretic and diuretic actions in the kidney (Gutkowska et al., 2014; Theilig and Wu, 2015), thereby reducing blood volume.

In the brain, ANP-mRNA is present in the hypothalamic suprachiasmatic nucleus, and ANP appears in both the SON and suprachiasmatic nucleus (Lipari et al., 2007). At the cellular level, ANP is present in and exocytotically released from membrane-bound vesicles as shown in cultured rat cortical astrocytes (Krzan et al., 2003; Chatterjee and Sikdar, 2013). Centrally released ANP can inhibit osmotically evoked VP and OT release through presynaptic inhibition of glutamate release from the OVLT. It has been reported that ANP application to rat hypothalamic explants did not affect depolarizing responses of VP neurons to local hypertonicity. However, ANP reversibly abolished the synaptic excitation of MNCs after hypertonic or electrical stimulation of the OVLT (Richard and Bourque, 1996). The inhibition of VP release following activation of ANP neurons can lead to diuresis, and decreased adrenocorticotropin release and blood pressure (Gutkowska et al., 1997). In addition, central ANP release is also increased by glucocorticoids (Lauand et al., 2007) and estradiol (Vilhena-Franco et al., 2011) in response to osmotic/volemic stimulation. Thus, astrocytic ANP can contribute to reducing hydromineral overload under various physiological states.

### Other Effects

Hormonal cross talks and receptor-receptor interactions are common phenomena in brain control of HB. For instance, estradiol acts mainly on the VP neurons in response to water deprivation, potentiating VP neuronal activation and VP secretion without altering VP mRNA expression (Vilhena-Franco et al., 2016). Bradykinin, thyrotropin-releasing hormone, neurotensin and opioids (Irazusta et al., 2001), as well as secretin (Bai et al., 2017) and prolactin (Seale et al., 2012), are also implicated in neurohumoral regulation of HB and their expressions are likely regulated by astrocytes. Lastly, astrocytes can participate in the synthesis of many hormones that, under different osmotic conditions, regulate osmolality by differential expression of peptidases involved in the maturation and degradation of peptide (pro)hormones and neuropeptides. For example, in water-loaded rats, prolyl endopeptidase was decreased in the brain cortex (Irazusta et al., 2001). Thus, astrocytes can participate in HB by catabolizing humoral factors as well. These lines of evidence indicate that astrocytes could contribute to the HB through multiple hormones.

## Osmosensation by Astrocytes

Homeostasis of the internal environment largely depends on neurohumoral regulation of the central osmosensory system. As reviewed recently (Jiao et al., 2017), the key feature of osmosensation is the activation of mechanoreceptors, particularly vallinoid and canonical types of transient receptor potential channels (TRPV and TRPC, respectively), both of which are highly permeable to Ca^2+^. Indeed, the activation of these TRP channels increases cytosolic Ca^2+^ levels in osmosensory cells, including VP neurons, and triggers a series of secondary reactions. For example, hypotonic stimulus induces intracellular Ca^2+^ elevations through TRP channels, which then trigger AQP1 translocation and activation (Conner et al., 2012). The activation of TRP channels relies on changes in cell volume, membrane stretch and cytoskeletal reorganization as well as the hydration status of the extracellular matrix (ECM) and activity of integrins in a spatiotemporal dependent manner. In this process, astrocytic plasticity plays a key role since acute hyperosmotic stimulus-induced Fos expression in neurons depends on activation of astrocytes in the SON of rats (Yuan et al., 2010). Moreover, disabling astrocytic plasticity also blocked the rebound excitation of VP neurons in response to hypoosmotic challenges (Wang et al., 2013a,b). Thus, astrocytes could be a prominent component of the osmosensory system and could be more sensitive to the osmotic challenges than neurons are.

### Characteristics of Osmosensation

Osmosensation is not a simple result of activation or inactivation of the stretch-activated channels (Cheng et al., 2017). To fully understand the participation of astrocytes in osmosensation, it is necessary to analyze the characteristics of osmosensation by the central osmosensory system.

#### Osmosensation Is Time-Dependent Process

In an organism, osmosensation is a dynamic process in temporal domain, as outlined below using two examples. (1) Chronic hyperosmotic stress weakens GABA_A_ receptor-mediated synaptic inhibition of VP and OT secretion in rat hypothalamic MNCs (Kim et al., 2011). That is, hyperosmotic stress caused a profound depolarizing shift in the reversal potential of GABAergic response (E_GABA_) in MNCs. This E_GABA_ shift was associated with increased expression of NKCC1 in MNCs and was blocked by the NKCC inhibitor bumetanide. (2) In response to hyposmolality challenge, VP neurons in the SON show a transient inhibition of firing activity followed by a rebound excitation (Wang et al., 2013a,b), which can partially account for the rebound increase in VP release after initial inhibition in response to hyponatremic challenges (Yagil and Sladek, 1990). It is likely that the initial osmosensory response involves Ca^2+^-activated K^+^ channels and, the BK and SK types of K^+^-selective ion channels (Ohbuchi et al., 2010), leading to elimination of action potentials and to hyperpolarization of membrane potentials, while the latter phase could be a result of RVD-related activation of the volume-regulated anion channel (VRAC) and Cl^−^ efflux (Muraki et al., 2007; Hübel and Ullah, 2016). Taken together, these findings indicate the presence of a diversity of mechanisms underlying temporal dynamics in osmosensation.

#### Osmosensation Is Related to the Spatial Location of Osmosensory Cells

Mechanosensitive ion channels could function differently at different loci. For instance, excitation of the OVLT neurons elicited by hyperosmotic stress was not affected by the deletion of TRPV 4 but was abolished in cells lacking TRPV 1 (Ciura et al., 2011). Thus, TRPV1 likely plays a dominant role in OVLT sensation of hyperosmotic stress; however, hyperosmolality-sensitive TRPV4 (Liedtke and Friedman, 2003) could carry out the same function at other loci. Moreover, same TRPV channels at different loci could have different sensitivity to osmotic challenges. For example, cell and cell nuclear sizes in the SON were increased or decreased with hyperosmolality or hypoosmolality, respectively. However, both conditions did not affect those sizes in the medial habenular nucleus *in vivo* (Zhang et al., 2001), although this brain area also expresses TRPV1 and TRPV4 (Ishikura et al., 2015). Furthermore, hyperosmotic glucose or urea solutions activate VP neurons but not CVO neurons (Ho et al., 2007). These findings indicate existence of spatial diversity in osmosensation.

#### Roles of Various Ion Channels in Osmosensation Might be Interchangeable

In classical view, sensation of osmotic pressure is the matter of activation or inhibition of stretch-activated cation channels, (Prager-Khoutorsky and Bourque, 2015). Besides the TRP channels discussed above, other mechanical ion channels have been implicated in osmosensation, such as TMEM63 proteins found in *Arabidopsis* (Zhao et al., 2016), acid-sensing ion channel 3 in the intervertebral disc (Uchiyama et al., 2007), mammalian TRP ankyrin-1 and TRP melastatin-8 channels (Soya et al., 2014). It is possible that once one ion channel is dysfunctional (Taylor et al., 2008; Ciura et al., 2011), other ion channels with similar responsive features could easily compensate this deficit and thus maintain the osmosensory ability.

### Osmoreceptors in Astrocytes

As stated above, the traditional theory of neuron dominance in osmosensation could not entirely explain findings presented above and, thus, the osmosensory ability of astrocytes needs to be considered in the operation of the osmosensory system.

#### AQP4 and Water Transport

Astrocytic volume change and subsequent morphological plasticity are essential for astrocytic regulation of local HB and VP neuronal activity (Theodosis et al., 2008; Wang and Zhu, 2014), the latter of which heavily relies on AQP4 expression and activity in astrocytes (Wang and Parpura, 2016).

In the rodent brain, AQP4 is the predominant form of specific water channel protein expressed in astrocytes; it is essential for quick water transport between intracellular and extracellular compartments when osmotic gradients are present (Xu et al., 2017). As previously reported, volemic change is essential for osmosensation as it changes the interaction between cytoskeletal elements and osmosensitive TRP channels (Prager-Khoutorsky and Bourque, 2015). Moreover, the expanded astrocyte processes promote releasing gliotransmitters and neuromodulators, metabolizing neural active substance, and transporting content of the interstitial fluid to the blood vessels (Parpura and Verkhratsky, 2012; Zorec et al., 2012) and thus exert the effect of osmosensation while maintaining the HB in the brain. The osmolality-associated AQP4 permeability determines astrocytic morphological plasticity, which can change the activity of their adjacent OS neurons independent of the activity of TRP channels, thereby making individual TRP channel type replaceable in osmosensation.

The participation of AQP4 in osmotic regulation is a dynamic process. It has been observed that the expression of AQP4 increased during chronic hyperosmotic stress (Yang et al., 2013). By contrast, hypoosmotic stimulation increases water influx through AQP4 due to increased osmotic gradient across cell membranes. This latter effect is not due to increase in AQP4 membrane installation but in its permeability to water (Potokar et al., 2013). It is possible, however, that there is a transient increase in AQP4 expression, or a reduction in its catabolism, at the early stage of hypoosmotic stimulation because levels of AQP4 are found to rapidly increase or decrease; these changes are accompanied by expansion and retraction of astrocyte processes in rat SON, respectively (Wang and Hatton, 2009).

#### Ion Channel Activity

Neurons in the CVOs and the MNCs in the SON and PVN, all important for thirst and urination, are also the target of astrocytic plasticity, which is related to alteration of astrocytic ion channel activity. In addition to the extensively identified astrocyte involvement in osmotic regulation of VP neuronal activity in the SON, astrocytes are also Na^+^ sensors. Namely, Na_x_, an atypical Na^+^ channel and Na^+^-level sensor, is found located in perineuronal lamellate processes extending from ependymal cells and astrocytes in the CVOs; astrocytes isolated from the SFO were sensitive to an increase in the extracellular Na^+^ level (Noda, 2007).

Another important osmosensory machinery in astrocytes is the expression of TRP channels. Astrocytes are known to express many types of osmosensitive TRP channels. For example, TRPV1 has been identified at thick cellular processes of astrocytes in the CVOs, and intravenous administration of a TRPV1 agonist resiniferatoxin induced Fos expression in astrocytes in the OVLT, SFO and area postrema (Mannari et al., 2013). The resultant Ca^2+^ influx following activation of TRP channels is not only associated with astrocytic volemic change but also related to neuromodulation through releasing glutamate, and this transmitter can modulate neuronal activity at extrasynaptic sites (Parpura et al., 2017). Thus, Na_x_ channels and TRPV1 channels in astrocytes can sense Na^+^ levels in bodily fluids (Noda, 2007), particularly in the blood, as well as in the interstitium of the osmotic regulatory system, and in turn convert hydromineral message to neuronal activity, thereby maintaining the HB. The different types of TRP channel expression in astrocytes and its association with different types of OS neurons endow some of the spatial features of the osmosensory system.

#### Osmolyte Transport and its Coupling to AQP4

Transporting osmolytes is essential for HB between intra- and extracellular spaces and involves the redistribution of osmolytes through ion transporters and channels.

The function of AQP4 is coordinated with a variety of closely associated osmolyte transporters. As reviewed recently (Wang and Parpura, 2016), AQP4 is colocalized and/or assembled with the TRPV4, Kir4.1/Kir5.1, connexin 43, glutamate transporter-1, metabotropic glutamate receptor 5, and Na^+^/K^+^-ATPase, mainly at astrocytic endfeet. Along with changes in AQP4 activity, there are also alterations in non-selective cation currents, K^+^ and Na^+^ transmembrane activity, and glutamate uptake and metabolism. In addition, many other molecules are also involved in this process, including: (1) channels, such as Ca^2+^ release-activated Ca^2+^ channel, voltage gated K^+^ channels, sulfonylurea 1/TRP melastatin-4 ion channel; (2) carriers including Na^+^, Cl^−^ cotransporter, NKCC, Na^+^/H^+^ exchangers, Cl^−^/HCO_3_^−^ exchanger, acid-sensing ion channel 1a, Na^+^-glucose cotransporter, and glutamate transporters; (3) receptor-coupled channels such as ionotropic AMPA and NMDA types of glutamate receptors, and GABA_A_ receptor; and (4) Na^+^/K^+^-ATPase or sodium pump, as reviewed recently (Jia et al., 2016). Lastly, epithelial Na^+^ channel (Miller and Loewy, 2013) and VRAC (Hussy et al., 2000) are also involved in astrocyte regulation of HB.

In astrocytes, there is NKCC-mediated Na^+^/K^+^/Cl^−^ inward flux evoked by elevated extracellular K^+^. The increase in clearance of extracellular K^+^ during neuronal excitation is initially performed by Na^+^/K^+^-ATPase-mediated K^+^ uptake into astrocytes; at K^+^ concentrations above ~10 mM this process is aided by uptake of Na^+^, K^+^ and 2 Cl^−^ via the cotransporter NKCC1. This activity allows subsequent K^+^ release via the inward rectifying K^+^ channel Kir4.1, perhaps after syncytial buffering (Walz, 1992), i.e., trans-astrocytic connexin-mediated K^+^ transfer. Since the Na^+^/K^+^-ATPase exchanges 3 Na^+^ with 2 K^+^, it creates extracellular hypertonicity and cell shrinkage; however, hypertonicity stimulates NKCC1 and can also cause RVI to minimize ionic disequilibrium due to asymmetric Na^+^/K^+^-ATPase fluxes (Hertz et al., 2013).

#### Astrocytic ECM

The ECM represents an essential component of osmosensation (Jiao et al., 2017). In response to hydromineral disbalance, the ECM can quickly detect changes in extracellular cation levels and then transmit this message to other components of the osmosensors, including integrins, cell adhesion molecules and cytoskeletal elements. Integrins can transduce hydromineral signals from extracellular to intracellular compartments by activation of TRP channels via integrin-linked kinase and actin, as previously discussed (Jiao et al., 2017). In the SON and PVN, neural cell adhesion molecule, which gets decorated with various amounts of sialic acid, can significantly influence cell adhesion and thus allows astrocyte processes to expand or retract (Theodosis et al., 2008).

The ECM-integrin bonding is modulated by both cell volume and hydration state of the ECM in osmotic stress. Under different osmotic conditions, signaling process modulated by integrin-ECM contacts is either activated or inhibited. As observed in the SON, hyperosmotic stress caused by increasing NaCl concentration can decrease polysialic acid-neural cell adhesion molecule complex content (Schmelzer and Miller, 2002) and increases extracellular volume (Perkins et al., 2017). The changes in the astrocytic ECM (Pierre et al., 2001) could further activate astrocytic TRP channels through integrins (Song and Dityatev, 2018). In contrast, their decrease promotes reactive gliosis or expansion of astrocytic processes that reduces neuronal excitability (Theodosis et al., 2008), at least transiently (Jiao et al., 2017). These features endow astrocytes with the ability to detect osmotic changes in the internal environment. It is likely that changes in extracellular osmolality activate TRP channels, which increases intracellular Ca^2+^, changes glial fibrillary acidic protein (GFAP) polymerization state and AQP4 distribution at the plasma membrane, leading to astrocytic morphological and functional plasticity.

#### GFAP Plasticity

The typical morphological feature of astrocytes during hydromineral disturbance is the plastic changes in the expression of GFAP, which determines not only the morphology, but also the function of astrocytes (Wang and Parpura, 2016). As the retraction and expansion of astrocytic processes take place, depolymerization/disassembling and polymerization/assembly of GFAP also occur, correspondingly; these events translocate GFAP-associated functional molecules, such as glutamine synthetase, AQP4 and actin, to (GFAP assembly) and from (GFAP disassembly) astrocyte processes surrounding adjacent neurons, thereby changing the uptake rate of extracellular neurochemical, their transport, conversion and/or release. As a result, neuronal activity is also changed as discussed below.

Hypotonic stimulation of the SON caused transient inhibition of VP neuronal activity (Wang et al., 2013a,b) and VP release (Yagil and Sladek, 1990), which were followed by recovery of the firing activity of VP neurons and rebound of VP secretion. These phenomena were accompanied with an initial increase in GFAP expression followed by a decrease in its expression, and were blocked by gliotoxin, L-aminoadipic acid. Importantly, GFAP increase was accompanied with simultaneous increase in the expression of glutamine synthetase and redistribution of this enzyme toward peripheral processes. This subcellular relocalization of glutamine synthetase to astrocytic processes (vs. soma) reflects upon an increased astrocytic ability to cause a shift in excitation-inhibition by decreasing extracellular concentrations of glutamate (as per increases conversion of glutamate to glutamine in astrocytes) and resulting in suppression of VP neuronal activity. Conversely a subsequent decrease in GFAP was associated with a decrease in membrane installation of GFAP-associated proteins and their functions (Wang et al., 2013a,b), including that of glutamine synthetase, resulting in an increase of extracellular levels of glutamate. Through this GFAP plasticity, astrocytes can adaptively exert their influence on the activity of VP and other OS neurons via differently localizing their functional proteins and changing neurochemical environment.

## Mechanisms Underlying Osmotransduction in Astrocytes

**Figure 2:**
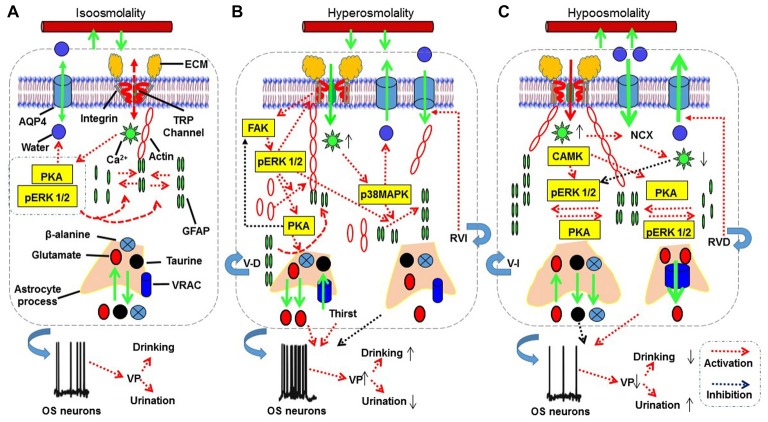
The proposed mechanisms underlying astrocytic sensation and transduction of osmotic signals. **(A–C)** The signaling processes for isosmotic **(A)**, hyperosmotic **(B)** and hypoosmotic **(C)** conditions, respectively. The abbreviations are: AQP4, aquaporin4; CAMK, calmodulin kinase; ECM, extracellular matrix; FAK, focal adhesion kinase; GFAP, glial fibrillary acidic protein; MAPKs, mitogen-activated protein kinases; NCX, sodium-calcium exchanger; OS neuron, osmosensory neurons; pERK 1/2, phosphorylated extracellular signal-regulated kinase 1/2; PKA, protein kinase A; RVD, regulatory volume decrease; RVI, regulatory volume increase; TRP, transient receptor potential; V-D, volume decrease; V-I, volume increase; VRAC, volume-regulated anion channel. See text for details.

Activation of osmosensory cells is only the beginning of the osmosensory reaction to hydromineral disturbance. Osmosensation in the osmosensory system initiates osmotransduction that is responsible for osmotic adaptation process. The osmotransduction involves a complex set of signaling processes that are responsible for astrocytic plasticity under osmotic stress (Figures 2A–C). In this process, changes in GFAP and AQP4 expression are critical events, linked to diverse signaling cascades.

### Transduction of Hyperosmotic Signals

Hyperosmotic stress can activate a variety of signaling molecules (Figure 2B). We provide a contemporary analysis of the spatiotemporal distribution of signaling molecules and their interactions; this analysis can aid better understanding of signaling cascades responsible for the transduction of different osmotic information in astrocytes. Several lines of evidence reveal that cellular effects of osmotic stress are commonly associated with intracellular Ca^2+^ mobilization and activation of mitogen-activated protein kinases (MAPKs) including c-Jun NH2-terminal kinase, p38 MAPK and extracellular signal-regulated kinase 1/2 (ERK 1/2; Figures 2A–C).

#### ECM-Integrin and Kinase Pathways

When osmotic stress acts on cell surface glycocalyx, integrin-mediated cell-matrix adhesion forms, cytoskeleton reorganizes, and focal adhesion kinase (FAK)-ERK1/2-c-Jun signaling axis gets activated, leading to the upregulation of interstitial collagenase, i.e., matrix metalloproteinase 13 (MMP-13) expression in rats (Horiguchi et al., 2011).

Hyperosmotic activation of the above pathway has dual effect on cellular events. The protective effect of integrins is clearly associated with phosphorylation/activation of FAK (Lunn and Rozengurt, 2004). In astrocytes, it has been reported that ECM binding to integrins can activate FAK, which is colocalized with actin stress fibers at sites of focal adhesion complexes (Rutka et al., 1999). An important downstream signal of FAK is phosphorylated ERK 1/2 (pERK 1/2) that is both essential for the activation of TRP channels during hyperosmotic stimulation (Prager-Khoutorsky and Bourque, 2015) and for GFAP polymerization. As reported, integrin-mediated ECM adhesions and cytoskeleton organization activate the FAK-ERK signaling axis, leading to upregulation of MMP expression and increased cell motility (Shi et al., 2011), a condition favoring astrocyte process retraction during hyperosmotic stimulation (Theodosis et al., 2008). Consistently, FAK signaling can promote organizing filamentous actin into parallel bundles (Rutka et al., 1999), which can interact with GFAP through calponin 3 (Plantier et al., 1999) and fascin (Mondal et al., 2010). Since an increase in this molecular association occurs during astrocyte retraction along with GFAP reduction (Wang and Hatton, 2009), the activation of FAK signaling could account for the astrocyte retraction following hyperosmotic stimulation, at least at the initial stage.

It is worth noting that increase in pERK 1/2 is essential for GFAP polymerization in the SON; however, pERK 1/2 is likely expressed in an undetectable level in astrocytes in the SON during hyperosmotic stimulation (Dine et al., 2014). Thus, the promoting effect of pERK 1/2 on GFAP polymerization could be minimal. Alternatively, activation of FAK-ERK 1/2 signaling could be restrained to the somata of astrocytes since pERK 1/2 promotion of GFAP polymerization has strong feature of microdomain-specificity (Wang et al., 2017). Lastly, pERK 1/2 signaling could be an upstream signal of protein kinase A (PKA) that can inhibit FAK activation (Padmanabhan et al., 1999) while increasing AQP4 permeability (Song and Gunnarson, 2012) and GFAP depolymerization in the SON (Wang et al., 2017).

It has been reported that cyclic adenosine 3′,5′-monophosphate (cAMP)-associated signaling is a component of the hyperosmotic signals. Salt-loaded rats had elevated levels of cAMP in the SON within 2 days of drinking 2% NaCl saline. Similarly, increasing medium osmolality from 290 to 325 mOsm/kg increased cAMP levels in the SON in rat explant culture (Carter and Murphy, 1989). Furthermore, increased cAMP promotes bovine VP gene expression (Pardy et al., 1992) through cAMP-PKA-CREB signaling (McCabe and Burrell, 2001). Albeit not directly demonstrated, one might expect that increased levels of cAMP would lead to increased expression-polymerization/assembly of GFAP in the SON, as it has been demonstrated in cultured astrocytes from mouse visual cortex (Gottipati et al., 2012). Taken together, it is very likely that a microdomain specific expression of pERK 1/2 and PKA is responsible for the GFAP-associated astrocytic plasticity during osmotic stress.

#### Cytosolic Ca^2+^ Levels and Associated Signaling Molecules

A direct effect of activating ECM-integrin signaling is the opening of TRPV channels, which causes increases in intracellular Ca^2+^ level. In turn, Ca^2+^ increase can activate MAPKs through Rho-type small G-protein (Kino et al., 2010), and osmotic stress further promotes couplings of activated Rho-type small G-proteins with c-Jun NH2-terminal kinase-interacting protein 4 to cascade components of the p38 MAPK signaling pathway. This signaling stimulates the expression of nuclear factor of activated T-cells 5, a transcription factor which regulates the expression of genes involved in the osmotic stress (Lee et al., 2011). Moreover, the activation of p38 MAPK also increases AQP4 expression (Salman et al., 2017), particularly following hyperosmotic stimulation (Arima et al., 2003), which facilitates water efflux from intracellular compartment to the extracellular space. Ca^2+^ signaling is also associated with GFAP plasticity (Chatterjee and Sikdar, 2013), which could also been mediated by p38 MAPK in addition to pERK 1/2 (Li et al., 2017). Indeed, in a mouse model of the middle cerebral artery occlusion, p38 MAPK activation was observed in the glial scar area, while in culture hypoxia and scratch injury-induced astrogliosis was attenuated by both p38 inhibition and knockout of p38 MAPK (Roy Choudhury et al., 2014); p38MAPK activation was associated with increased GFAP expression. Since increase in GFAP expression along with its associated astrocytic processes expansion does not occur at the initial stage of hyperosmotic stimulation, the GFAP increase could be a secondary reaction occurring before the RVI. Lastly, Ca^2+^ signaling can contribute to the RVI by antagonizing a PKA-mediated increase in AQP4 permeability (Song and Gunnarson, 2012), which is supposed to further reduce water loss in the presence of higher extracellular osmolality. However, detailed regulation of GFAP reduction and astrocyte retraction following hyperosmotic stimulation remains to be explored.

#### Pro-inflammatory and Apoptotic Pathway

Chronic and severe hyperosmotic challenges can cause maladaptation of astrocytes and consequently result in detrimental responses of the osmosensory system. Uncontrolled hyperosmotic stress-elicited TRPV1 channel activation increases release of cytokines interleukin-6 and interleukin-8 which could result from transactivation of epidermal growth factor receptor, MAPK, and nuclear factor kappa-light-chain-enhancer of activated B cells activation (Cavet et al., 2011). Moreover, hyperosmotic saline also acts via MMP9 that binds to the low-density lipoprotein receptor-related protein-1, triggers the phosphorylation of ERK 1/2, and induces down-regulation of the perineurial barrier-forming tight junction protein claudin-1 (Hackel et al., 2012). The activation of MAPKs can also evoke cell apoptosis. For example, hyperosmotic stress-induced apoptosis is mediated by p38 MAPK, c-Jun NH2-terminal kinase and activating AP-1 transcription complex, and manifests with acceleration of cytochrome c release and caspase-3 activation (Ben Messaoud et al., 2015). Thus, hyperosmotic stress could cause systemic inflammation and apoptosis if dehydration or hypertonic stress is not corrected promptly.

### Hypoosmotic Signals

Under hypoosmotic challenges, cell swelling and the ensuing RVD could involve a series of cellular and molecular events, which lead to adaptive changes in astrocytic morphology (Figure 2C).

#### Cytosolic Ca^2+^, pERK1/2 and GFAP Plasticity

Hypoosmolality initially causes mild cell swelling that activates Ca^2+^-permeable cation channels such as TRPV 4 (Benfenati et al., 2007) and leads to transient Ca^2+^ influx into the cells (Sato et al., 2013). The Ca^2+^ influx along with increased membrane stretch triggers Ca^2+^ release from intracellular stores (Borgdorff et al., 2000). This elevation in intracellular Ca^2+^ is essential for an increase in formation of GFAP filaments (González et al., 2007; Salazar et al., 2008) and a hypoosmotic volume increase (Ebner et al., 2006). Hypoosmolality-elicited Ca^2+^/calmodulin signaling can increase the expression of pERK1/2 that is likely responsible for Ca^2+^-associated cell swelling because chelation of extracellular Ca^2+^ also abolished pERK 1/2 increases under hypoosmotic conditions, while blocking the phosphorylation of ERK 1/2 significantly reduced cell swelling and the ensuing RVD, as shown in trout hepatocytes (Ebner et al., 2006). Importantly, prolonged hypoosmotic challenge *in vitro* was found to evoke only transient intracellular Ca^2+^ increase that was followed by a long decrease in Ca^2+^ levels (Sánchez-Olea et al., 1993), likely due to the activation of plasmalemmal Na^+^-Ca^2+^ exchangers as shown in mouse odontoblasts (Sato et al., 2013). In the SON, the pERK1/2-associated GFAP polymerization and the elongation of astrocytic processes (Wang et al., 2017) appears as a short-lasting (minutes) event, which could be reversed by some subsequent event that causes GFAP depolymerization, such as PKA activation (Wang et al., 2017). The causal association between GFAP depolymerization and hypoosmolality-evoked RVD in chronic hypoosmotic challenges remains to be elucidated.

#### Cellular Signaling and RVD

Following Ca^2+^ influx and mobilization of intracellular Ca^2+^ store, high cytosolic Ca^2+^ levels lead to further cell swelling and RVD (Mola et al., 2015). In this process, calmodulin kinase plays a key role since intracellular application of monoclonal anti-calmodulin antibody blocked hypoosmotic activation of VRAC opening in rat cerebral astrocytes (Olson et al., 2004). Moreover, p38 MAPK is involved in the occurrence of RVD in hypoosmotic environment (Ebner et al., 2006). Whether these signals are directly linked to RVD remains to be explored.

Interestingly, PKA activation is associated with increased activation of VRAC and efflux of taurine and Cl^−^ from astrocytes with redistribution of actin network, i.e., its disruption at the somata, while concentrated at foci corresponding to the tips of the cell projections retracted by swelling (Moran et al., 2001). Moreover, actin cytoskeleton and microtubule are important for astrocyte swelling and they may form a barrier for the efflux of taurine and Cl^−^ since the presence of dense actin network can impede the flow of ions and water (Lange, 2000; Platonova et al., 2015).

#### Effects of Hypoosmotic Signals on Neuronal Activity

Accompanying the above signaling events and the resultant morphological changes, astrocytes could change neuronal activity through releasing gliotransmitters. The initial hypoosmotic inhibition of VP neuronal activity is associated with the increased availability of extracellular β-alanine, presumably through the reduction in its uptake (Johnston and Stephanson, 1976); the increased level of this non-essential amino acid then inhibits plasmalemmal GABA transporters and subsequently increases extracellular GABA levels (Park et al., 2006). The rebound excitation of VP neurons during hypoosmolality-evoked RVD is associated with the failure of GABAergic inhibition (Wang et al., 2013a) and coordinated D-serine signaling between astrocytes and MNCs in the SON to increase both NMDA receptor activation and VP neuronal activity (Wang et al., 2013b). In addition, the release of ATP from astrocytes during cell swelling could also contribute to the rebound excitation of neurons that are initially inhibited by hypoosmolality (Förster and Reiser, 2016) by activating ATP-sensitive K^+^ channel (Thomzig et al., 2003). Lastly, hypoosmotic challenge-evoked taurine release through VRAC is also involved in the transmission of osmotic information. In the SON, taurine is released from astrocytes in an osmolality-dependent manner (Choe et al., 2012) and acts on glycine receptors to inhibit VP neuronal activity (Hussy et al., 2000). However, this effect is reversible during prolonged hypoosmotic challenges (Song and Hatton, 2003). The summary of potential mechanisms underlying astrocyte sensation and transduction of osmotic signals are outlined in Figure 2.

## Astrocytes and Intracellular Cerebral Edema

Cerebral edema is a common outcome when excess fluid is accumulated in the intracellular or extracellular space of the brain due to brain trauma or hydromineral disorder. According to the extent of the BBB disruption, cerebral edema can be divided into two types: (1) Cerebral edema with BBB disruption, which includes both vasogenic and interstitial edemas, featuring extracellular retention of water and Na^+^; and (2) Cerebral edema without the BBB disruption, which includes cytotoxic and osmotic edemas, both of which feature intracellular swelling. Astrocytic plasticity is involved in brain edemas. Pathogenesis of vasogenic and interstitial edemas was recently reviewed (Jia et al., 2016; Wang and Parpura, 2016), so was the involvement of maladaptive ion transport in the occurrence of cerebral edema (Stokum et al., 2016); ionic edema is usually associated with cytotoxic edema, where water and ions exit capillaries into the brain interstitium. Thus, in this review we further explore the mechanisms underlying astrocyte-associated cytotoxic edema and osmotic edema.

### Hyponatremia and Cerebral Edema

Hyponatremia is the most common clinical electrolyte disorder. When Na^+^ plasma concentration falls more than several mmol/L below normal level, hyponatremia occurs (Kleindienst et al., 2016), and is associated with decompensated heart failure, hepatic cirrhosis with ascites formation, renal failure, inappropriate intravenous transfusion, and cancer-associated syndrome of inappropriate antidiuretic hormone secretion. Hyponatremia due to loss of NaCl occurs as a consequence to diarrhea and vomiting, overuse of diuretics, certain types of Na^+^-wasting kidney diseases and Addison’s disease with reduced aldosterone (Muñoz et al., 2010; Raimann et al., 2017). Hyponatremia creates an abnormal osmotic pressure gradient across the BBB and plasma membrane, which drives movement of water into the brain parenchyma and leads to osmotic cerebral edema (Walcott et al., 2012; Voets and Maas, 2018). The subsequent water intoxication leads to astrocytic swelling and RVD, which further disturb the activity of osmotic regulating system.

Hyponatremia-evoked cerebral edema likely delivers signals through the following process. As reported, excessive Ca^2+^ influx through TRPV4 predisposes Müller glial cells of the retina to activation of Ca^2+^-dependent proapoptotic signaling pathways (Ryskamp et al., 2011). In glial cells of nematodes, it was also identified that osmotic stress induces massive protein aggregation coupled with unfolded protein response and endoplasmic reticulum (ER) stress (Gankam-Kengne et al., 2017); adequate protein folding is a tightly regulated process that requires proper intracellular ionic strength and it is necessary for normal cell function. Prolonged hyponatremia along with the activation of proapoptotic pathways could similarly cause damages to mammalian astrocytes. During RVD, the reversal of glutamate transporters and anion channel opening could increase glutamate release (Milanese et al., 2009). A sustained low extracellular sodium ion concentration decreased glutamate uptake by astrocytes and elevated extracellular glutamate concentration (Fujisawa et al., 2016). The release of glutamate from astrocytes can activate extrasynaptic NMDA receptors (Araque et al., 1998) to increase spontaneous synaptic transmission which can lead to an increase in production of NO (Kenny et al., 2018). Prolonged accumulation of NO could cause protein tyrosine nitration (PTN), damage the oxidative phosphorylation and trigger apoptosis in astrocytes (Raju et al., 2015). As a result, astrocyte uptake mechanism, e.g., for glutamate, can be disrupted, leading to the accumulation of the extracellular glutamate, and consequently increased VP neuronal activity and VP secretion as previously reviewed (Wang et al., 2011; Jia et al., 2016). However, since PTN is only possible with NO levels way in excess of what can be expected from either neuronal or epithelial NOS under strong hyperosmotic stimulus, the PTN-associated apoptosis in astrocytes is most likely a result of activation of inducible NOS as discussed in the pathogenesis of cardiac injury (Wang et al., 2018).

It is important to note that MNCs in the SON remain functional during dehydration and ischemic stroke because of down-regulation of NMDA glutamate receptor (Currás-Collazo and Dao, 1999), and neuroprotection by glia and vascular/perivascular cells (Currás-Collazo et al., 2002).

### Central Pontine Myelinolysis

Central pontine myelinolysis is an osmotic demyelization syndrome often occurring during overly rapid correction of chronic hyponatremia with hypertonic saline infusion or using VP receptor antagonist. Astrocytes play an essential role in its development. As reported in rat models, massive astrocyte death occurred after rapid correction of hyponatremia. Astrocyte death caused a disruption of the astrocyte-oligodendrocyte network, rapidly upregulated inflammatory cytokines genes, and increased serum S100β, which predicted clinical manifestations and outcome of osmotic demyelination (Gankam Kengne et al., 2011). Treatment of hyponatremia with non-peptide VP receptor antagonists (vaptans) or hypertonic saline resulted in a higher mortality rate, when compared to treatment with urea, because of osmotic breakdown of the BBB, microglia activation, astrocyte demise and demyelination (Gankam Kengne et al., 2015). Moreover, astrocytes in central pontine myelinolysis were small, and exhibited fewer and shorter processes than perilesional astrocytes (Popescu et al., 2013) in addition to the loss of AQP4 in astrocytes within demyelinating lesions (Takagi et al., 2014). The damage of astrocytes is associated with imbalance between protein synthesis and degradation which can induce ER stress and cell death. As reported, rapid correction of chronic hyponatremia induces severe alterations in proteostasis, i.e., the biogenesis, folding, trafficking and degradation of proteins present within and outside the cell. The alterations are characterized by diffuse protein aggregation, ubiquitination and ER stress accompanied by increased autophagic activity and apoptosis in astrocytes within regions previously shown to be demyelinated in later stages of this syndrome (Gankam-Kengne et al., 2017). These results indicate that osmotic demyelination during severe osmotic stress might be a consequence of the failure of proteostasis. Furthermore, these results support a model for the pathophysiology of osmotic brain injury in which rapid correction of hyponatremia triggers apoptosis in astrocytes followed by a loss of trophic communication between astrocytes and oligodendrocytes, secondary inflammation, microglial activation and finally demyelination.

### Ischemic Cerebral Edema

The cytotoxic cerebral edema results from disruption in cellular metabolism and cellular retention of sodium and water due to poisoning or hypoxia. Hypoxia-associated cytotoxic cerebral edema is often observed in ischemic cerebral stroke (Jia et al., 2016), traumatic brain injury (Burda et al., 2016), cerebral palsy (Thomas et al., 2004) and cardiac arrest (Hirko et al., 2008). Astrocytes are critical contributors in this process, as typically observed in ischemic stroke (Wang and Parpura, 2016).

In the ischemic cerebral edema, a disruption of ionic and neurotransmitter homeostasis plays a pivotal role, particularly the accumulation of K^+^ and glutamate in the extracellular space. The activation of astrocytic NKCC1 by highly increased extracellular K^+^ concentrations and hypoxic inhibition of Na^+^/K^+^-ATPase form conditions promoting influx of Na^+^/K^+^/Cl^−^, while inhibiting efflux of K^+^ and Cl^−^ through K^+^ and Cl^−^ co-transporter (Wilson and Mongin, 2018). During reperfusion following initial ischemia in the stroke, with the activation of Na^+^/K^+^-ATPase, more Na^+^ release than K^+^ uptake occurs in a 3:2 ratio, which drives NKCC1 to move more ions into the cell, leading to the development of cytotoxic cell swelling (Wang and Parpura, 2016). All these pathological processes are associated with malfunctioned uptake machinery for K^+^ and glutamate (Steiner et al., 2012; Yan et al., 2016) and fluid volume transfer through AQP4 (Anderova et al., 2014) by astrocytes during ischemic stroke. Additionally, there is an increased level of extracellular VP during ischemic stroke (Jia et al., 2016). This notion is supported by a clinical observation that continuous intravenous infusion with conivaptan, a non-peptide antagonist of V1a and V2 VP receptors, for 48 h after experimental stroke reduces brain edema and BBB disruption (Zeynalov et al., 2015). Thus, in this process, damaged astrocytes and malfunctioned VP neurons could form a vicious cycle to worsen ischemic brain damages.

The disruption of ionic/neurotransmitter homeostasis is derived from reduced delivery of nutrients to the brain, in particular glucose and oxygen. In this process, disorders of many interacting molecular pathways in astrocytes, particularly TRPV2 activation-associated Ca^2+^ overload, are implicated. As reported in rat cortical astrocytes, oxygen-glucose deprivation followed by reoxygenation treatment enhanced the expression of TRPV2 and increased intracellular Ca^2+^ level (Zhang et al., 2017), which could disrupt normal functions involving mitochondria, ER, and nucleus, leading to cell damage.

Under physiological conditions, mitochondrial activity depends on increases in cytosolic Ca^2+^, from the extracellular space or from the ER, which is taken up by mitochondria through specialized contact sites between the ER and mitochondria known as mitochondrial-associated ER membranes. The coordination of these Ca^2+^ pools is required to synchronize mitochondrial respiration rates and ATP synthesis to meet physiological demands (Schäfer et al., 2014). However, serious hypoxia and glucose deprivation during ischemic stroke could cause strong mobilization of intracellular Ca^2+^ stores, ER stress, mitochondrial Ca^2+^ overload and oxidative stress in astrocytes (Hori et al., 2002; Ouyang et al., 2011), leading to inflammatory reaction and apoptosis. In this process, abnormal RVD following initial astrocytic swelling could create a hyperosmotic environment (Wang and Parpura, 2016) to cause inflammation (Cavet et al., 2011) and astrocytic apoptosis (Ben Messaoud et al., 2015), leading to irreversible brain damage.

**Figure 3:**
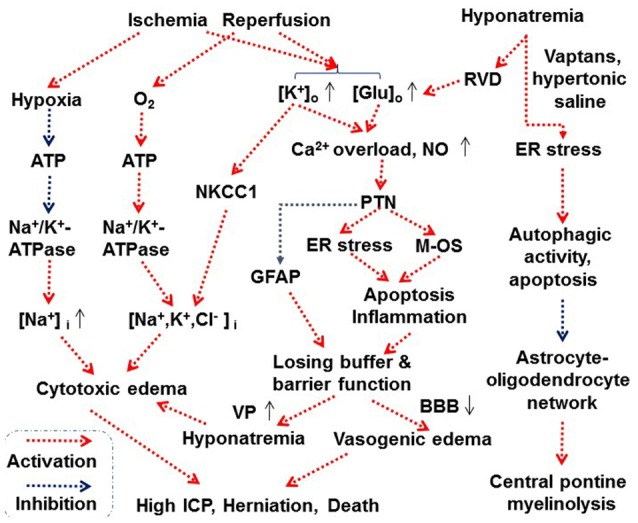
Scheme depicting proposed astrocytes-associated mechanisms underlying cerebral edema. Abbreviations: BBB, blood-brain barrier; ER, endoplasmic reticulum; Glu, glutamate; ICP, intracranial pressure; M-OS, mitochondrial oxidative stress; NKCC1, Na^+^, K^+^, 2 Cl^−^ and water cotransporter 1; NO, nitric oxide; PTN, protein tyrosine nitration. See text for details. Other annotations refer to Figures 1, 2.

It is worth to notice that a knockout of AQP4 yielded contradictory reports, some claiming reduction in post-traumatic edema (Shi et al., 2012; Wu et al., 2014) while the others just the opposite Hirt et al. (2017). It is possible that AQP4 is differentially involved in different types of cerebral water balance. By slowing the rate of water entry into brain, AQP4-null mice could be protected from cytotoxic brain edema induced by water intoxication, brain ischemia, or meningitis. By contrast, by reducing the rate of water outflow from brain parenchyma, AQP4 deletion could aggravate vasogenic brain edema caused by tumor, cortical freeze, intraparenchymal fluid infusion, or brain abscess (Papadopoulos and Verkman, 2007). However, the detailed mechanisms underlying this controversy remain to be explored. Figure 3 summarizes the mechanisms underlying astrocytes-associated cerebral edema.

## Conclusion

Malfunctions of astrocytes have been observed in many diseases in addition to those that are directly associated with hydromineral disorders, such as epilepsy (Losi et al., 2016), neuroinflammatory demyelinating disease (Verkman, 2012), Canavan disease (Clarner et al., 2014), Huntington’s disease (Hsiao et al., 2014), amyotrophic lateral sclerosis (Ramírez-Jarquín et al., 2017), and post-traumatic syringomyelia (Najafi et al., 2016). Understanding of the involvement of astrocytes in neurohumoral modulation of HB and astrocytic mechanisms underlying cerebral edema will provide important references for clarification of all the pathogenesis of these diseases. Consequently, specific therapeutic targets might be identified with the perspectives of prevention of disease development and improvement of their prognosis.

## Author Contributions

Y-FW wrote the first draft. Y-FW and VP designed this topic and edited the manuscript.

## Conflict of Interest Statement

The authors declare that the research was conducted in the absence of any commercial or financial relationships that could be construed as a potential conflict of interest.

## Abbreviations

Ang II: angiotensin II
ANP: atrial natriuretic peptide
AQP 4: aquaporin 4
BBB: blood-brain barrier
cAMP: cyclic adenosine 3′,5′-monophosphate
CVOs: circumventricular organs
ECM: extracellular matrix
ER: endoplasmic reticulum
ERK 1/2: extracellular signal-regulated kinase 1/2
FAK: focal adhesion kinase
GABA: γ-aminobutyric acid
GFAP: glial fibrillary acidic protein
HB: hydromineral balance
I/R: ischemia/reperfusion
MAPKs: mitogen-activated protein kinases
MNCs: magnocellular neuroendocrine cells
NCX: sodium-calcium exchanger
NKCC1: Na^+^, K^+^, 2 Cl^−^ and water cotransporter 1
NO: nitric oxide
NTS: nucleus of the tractus solitarii
OT: oxytocin
OVLT: organum vasculosum of the lamina terminalis
pERK 1/2: phosphorylated ERK 1/2
PKA: protein kinase A
PTN: protein tyrosine nitration
PVN: paraventricular nucleus
RAAS: renin-androgen-aldosterone system
RVD: regulatory volume decrease
RVI: regulatory volume increase
SFO: subfornical organ
SON: supraoptic nucleus
TRP channels: transient receptor potential channels
TRPC: TRP canonical channel
TRPV: TRP vallinoid channel
VP: vasopressin
VRAC: volume-regulated anion channel.
